# Associations between Self-Rated Health and Perinatal Depressive and Anxiety Symptoms among Latina Women

**DOI:** 10.3390/ijerph191911978

**Published:** 2022-09-22

**Authors:** Janeth Juarez Padilla, Chelsea R. Singleton, Cort A. Pedersen, Sandraluz Lara-Cinisomo

**Affiliations:** 1 Division of Scholarship and Research, Columbia University School of Nursing, New York, NY 10032, USA; 2 Department of Social, Behavioral, and Populations Sciences, Tulane School of Public Health and Tropical Medicine, New Orleans, LA 70112, USA; 3 Department of Psychiatry, School of Medicine, University of North Carolina at Chapel Hill, Chapel Hill, NC 27599, USA; 4 Department of Kinesiology and Community Health, College of Applied Health Sciences, University of Illinois Urbana-Champaign, Champaign, IL 61820, USA

**Keywords:** self-rated health, perinatal, depression, anxiety

## Abstract

Purpose: The objective of this study was to determine whether decreases in or consistently low preconception to pregnancy self-rated health (SRH) were associated with perinatal depressive and anxiety symptoms among Latinas. Methods: This is a secondary data analysis of 153 perinatal Latinas. Three groups were created to capture SRH from preconception to pregnancy: a decline in ratings, consistently low, and good+ (i.e., good, very good, or excellent). SRH was measured using two questions about their perceived physical health before and during pregnancy. Depressive symptoms and anxiety symptoms were assessed in the third trimester and six weeks postpartum using the Edinburgh Postnatal Depression Scale and State-Trait Anxiety Inventory, respectively. Life stressors were assessed in pregnancy using a modified version of the Life Experiences Survey. Linear regressions tested the associations. Results: Women with consistently low (i.e., fair or poor) SRH reported significantly more prenatal depressive symptoms than women who reported consistently good+ SRH. Women who reported a decline in SRH to fair or poor reported more prenatal anxiety symptoms but decreased postpartum anxiety symptoms than women who reported consistently good+ ratings. Life stressors were positively associated with prenatal depressive and anxiety symptoms. Conclusions: Healthcare practitioners should assess changes in SRH ratings to identify risks for prenatal depressive and anxiety symptoms among Latinas, who have elevated rates of depressive and anxiety symptoms compared to non-Hispanic White women. Policymakers should provide healthcare providers with mental health resources to support at-risk Latinas during the prenatal period.

## 1. Introduction

The perinatal period has been associated with an increased risk for depression and anxiety [1,2]. The American College of Obstetricians and Gynecologists defines perinatal depression as any major or minor depressive episode with onset during pregnancy or in the first year after childbirth [3]. In the U.S., perinatal depression affects over 12–19% of women [4,5], with Latina women reporting higher rates than non-Latina White women [6,7]. Consequently, Latinas in the U.S. are at higher risk for depression and anxiety than non-Latina women, with rates of 60% and 19.4% in the perinatal period, respectively [7,8,9]. 

Perinatal anxiety includes the presence of anxiety during pregnancy to the first postpartum year [10]. In the U.S., the rate of perinatal anxiety has been estimated to be between 21 and 24% during pregnancy and 2–45% postpartum [11,12,13]. Research has shown that anxiety and depression can be comorbid [14,15]. The literature suggests Latina women report more anxiety during pregnancy than non-Latina White women [16]. Given the high rates of depression and anxiety in Latinas, it is critical to identify risk factors to develop prevention and intervention measures.

Self-rated health (SRH), commonly measured on a scale from poor to excellent, is a predictor of premature mortality, and those with poor SRH can be vulnerable to premature mortality [17]. SRH is a subjective evaluation of an individual’s health condition and has been shown to be a reliable and valid measure of perceived health among various populations [18,19,20]. Depressive symptoms can negatively affect women’s perception of their health during vulnerable critical periods such as pregnancy and postpartum. Poorer SRH ratings have been observed in prenatal and postpartum women compared to ratings before pregnancy [21,22]. Poor mental health can negatively affect SRH during the perinatal period [23,24,25,26]. One study showed that Latina women rated their health during pregnancy worse than preconception, and Latina women with prenatal depression reported worse SRH [27]. However, few studies have examined the changes in SRH over the perinatal period and its association with anxiety and depressive symptoms [20,28], including perinatal Latina women who have high experience high rates of these symptoms and complex stressors. 

Latina women are an important group to consider because they have high rates of economic stress, poverty, low education, discrimination, and immigrant-related stress [29,30]. For example, perinatal Latina women report experiencing more discrimination during pregnancy and postpartum than non-disadvantaged women in the U.S. [30]. Perinatal Latinas also have less access to medical care and, consequently, lower use of prenatal care [31]. Therefore, it is important to the role of psychosocial stressors when examining the association between SRH and depression or anxiety in perinatal Latinas [10,25].

The objective of this study was to determine whether perinatal depressive and anxiety symptoms were associated with changes in SRH in a sample of Latina women living in the U.S. We focused on changes in SRH from preconception to pregnancy, paying particular attention to women whose ratings declined to poor or fair or whose ratings remained low (i.e., poor or fair) because the research shows that these ratings are associated with increased risk for poor mental and physical health [32,33]. This study is novel because, to our knowledge, it is one of the first to assess changes in SRH ratings and perinatal anxiety and depressive symptoms, whereas others have focused on “poor” ratings among Latina women. This study is guided by two research questions: (1) Is there a significant change in SRH from preconception to pregnancy? and (2) Is a decline in SRH, defined as a “fair” or “poor” from “excellent”/“very good”/”good” associated with prenatal or postpartum depressive and anxiety symptoms? We hypothesized that women whose SRH ratings declined from preconception to pregnancy would report more depressive and anxiety symptoms in pregnancy and six weeks postpartum than those whose ratings remained high (i.e., “good, very good, or excellent” or did not decline to fair/poor). We also hypothesized that women with consistently low ratings (i.e., fair or poor) from preconception to pregnancy would report more depressive and anxiety symptoms in pregnancy and at six weeks postpartum than those whose ratings remained high (i.e., “good, very good, or excellent”) or did not decline to fair/poor.

## 2. Materials and Methods

### 2.1. Data Source and Study Participants

This cross-sectional study uses secondary data from a longitudinal parent study conducted between July 2008 and August 2012 in Raleigh, North Carolina [34]. In the parent study, 325 women at 31 to 33 weeks gestation were recruited from a local public health clinic and were enrolled at 35–36 weeks. Women were excluded from the parent study if they had: a lifetime history of bipolar, cyclothymic, psychotic, somatoform, or dissociative disorders according to the Diagnostic and Statistical Manual of Mental Disorders, Fourth Edition, were unable to write or speak English or Spanish, had a body mass index > 35 or < 18, had some experience with acute or chronic serious medical illness or pregnancy complication [34]. Two-hundred and sixteen (66.5%) had complete data for the longitudinal study. Of the 216, 153 self-identified as Hispanic/Latina and were included in this cross-sectional study. Participants who did not identify as Hispanic or Latina were excluded from this study. Data for the current study are based on the prenatal visit and six-week postpartum interview. The data were collected in participants’ preferred language (Spanish or English). Participants provided written consent to participate in the parent study. All the measures were available in English or Spanish. The study was conducted according to the guidelines of the Declaration of Helsinki and approved by the Institutional Review Board of the University of North Carolina at Chapel Hill. 

### 2.2. Measures

#### 2.2.1. Demographic Characteristics

Age, family income, and education were collected in the demographic questionnaire during the prenatal interview. Family income was pre-categorized into the following groups: less than USD 10,000, USD 10,001–20,000, USD 20,001–30,000, and more than USD 30,000. Education level was also pre-categorized as high school or less and college or greater, with college or greater. Demographic characteristics were used as covariates in the models.

#### 2.2.2. Self-Rated Health (Predictor Variable)

During pregnancy, women answered two questions about their physical health. (1) Compared to other people your age, how would you describe the state of your physical health, during the year before your pregnancy? and (2) Compared to other people your age, how would you describe the state of your physical health, since you’ve been pregnant? The two questions were derived from the Short Form Health Survey 12 [35], which measured self-rated global health. The Short Form Health Survey 12 questions focus on one’s perceived health, such as: “Have you felt calm and peaceful?” and “Did you have a lot of energy?”. Responses are based on a 5-point scale ranging from “poor” (5) to “excellent” (1). For this study, responses were reverse-coded as follows: “poor” = 1, “fair” = 2, “good” = 3, “very good” = 4, and “excellent” = 5. This study used the question about self-rated health. These measures of SRH have been used in previous studies [17,27,36] and one study including a sample of prenatal Latinas (Lara-Cinisomo, Swinford et al., 2018). The Cronbach’s α for SRH was 0.65, which indicated low internal consistency.

Three groups were created to determine associations with SRH: decline, consistently low, and good+. The decline group includes women whose SRH declined to either “poor” or “fair” from before preconception to pregnancy. The consistently low group represents women who experience no change in their “poor” or “fair” ratings from pre-conception to pregnancy. The good+ category included the rest of the sample and was labeled the reference group. These women consistently rated SRH as very good, good, or excellent between preconception and pregnancy. Changes in SRH ratings were grouped to consistently low decline, and good+ to learn more about the association between perinatal depressive and anxiety symptoms and declining SRH and consistently low SRH.

#### 2.2.3. Life Stressors as Covariate

A modified version of the Life Experiences Survey (LES) [37] was administered during pregnancy. The 29-item scale assessed the number of stressful life events during the last six months and how stressful the event was for the woman. Women indicated whether events such as death or a serious illness to a close family member, child, or parent occurred using yes (1) or no (0) responses for a total of 32 events. For the study, we only focused on the total number of life stressors, with a higher score indicating a higher number of stressful events. Life stressors were modeled as a covariate. Our sample reported zero to eight stressful life events during pregnancy. The modified LES has been demonstrated to be reliable and valid, and previous studies have shown a high internal consistency (Cronbach’s α = 0.58–0.88) [38,39]. The LES has been used among low-income and young Latina women [40,41].

### 2.3. Outcome Variables

#### 2.3.1. Depressive Symptoms

At 35–36 weeks gestation and six weeks postpartum, women completed the Edinburgh Postnatal Depression Scale (EPDS) [42] to determine the presence of depressive symptoms. The EPDS is a 10-item screener used to determine how the participant has felt in the last seven days before the assessment. Items are scored from 0 to 3, with some items reverse-scored. Scores range from 0 to 30, with a cutoff score of ≥10 for possible depression [42]. The EPDS has also been used in previous studies with low-income and Spanish-speaking Latina women [43,44]. It has also been shown to be a reliable tool to assess depressive symptoms, and previous studies have shown a high internal consistency (Cronbach’s α = 0.88–0.91), including when used with Latinas [45,46]. The EPDS score was used as a continuous outcome variable.

#### 2.3.2. Anxiety Symptoms

Participants also completed the 20-item trait portion of the State-Trait Anxiety Inventory (STAI-T) to assess anxiety symptoms [47] in pregnancy and six weeks post-childbirth. The trait portion represents how the individual generally feels daily, and has been used among low-income Spanish-speaking Latinas [44,48]. For each item, participants’ self-ratings were “almost never” = 1, “sometimes” = 2, “often” = 3, and “almost always” = 4. Total scores ranged from 20 to 80, with higher scores indicating higher anxiety [47]. High anxiety is often considered a score of 40 or more [49]. The STAI has been demonstrated to be a valid and reliable tool for anxiety in the prenatal and postpartum period, and previous studies have shown a high internal consistency (Cronbach’s α = 0.86–0.96) when used with perinatal women [49,50]. STAI-T has been used in studies with low-income, Spanish-speaking Latina women [27]. In this sample, STAI-T scores were used as a continuous outcome variable.

### 2.4. Statistical Analysis Plan

Descriptive statistics were calculated for all variables, with mean and standard deviations for continuous variables and percentages for categorical variables. Depression and anxiety scores during pregnancy and six months postpartum were the primary outcome variables. Data for outcome variables were reviewed for normality and outliers. The skewness of the outcome measures was closer to ± 1.0 and thus not considered normal. Because the sample was small and not normally distributed, nonparametric tests were performed. Means and standard deviations were provided for the outcomes of interest. Wilcoxon signed-rank tests were conducted to determine if there were differences between prenatal to postpartum depressive and anxiety symptoms.

Crude and multiple linear regressions with bootstrapping were used to determine whether SRH was associated with perinatal depressive and anxiety symptoms. Four bootstrap models with 1000 samples were fitted for each outcome measure. Model 1 represents the crude linear regression model that tested the association between SRH and depressive and anxiety symptoms during pregnancy and at six weeks postpartum. Subsequent models controlled for demographic characteristics and prenatal symptoms when testing six-week outcomes. The final model included all tested variables and life stressors Statistical analyses were performed using SPSS version 25.0 software using a significance level of 0.05.

## 3. Results

### 3.1. Sample Characteristics

The mean age of the sample was 26.88 years (SD = 5.41), 90.2% had less than a high school education, and 39.2% had a family income between USD 10,001 and 20,000 (shown on Table 1). Fifteen percent of the sample perceived their health as “fair,” and 1.3% reported “poor” health before pregnancy. Approximately 19.6% perceived their health as “fair” and 2% as “poor” since their pregnancy. The median EPDS scores were 7 at prenatal and 4.50 at six weeks postpartum. The median STAI scores during pregnancy were 36 and 30 at six weeks. The Wilcoxon signed-rank test indicated that depressive symptoms in pregnancy declined at six weeks postpartum (Z = −3.33, *p* = 0.001). Similarly, anxiety symptoms at pregnancy decreased at six weeks postpartum (Z = −5.22, *p* < 0.001) (shown in Table 2). The Wilcoxon signed-rank test also indicated that SRH declined from pregnancy to six weeks postpartum (Z = −3.22, *p* = 0.001).

### 3.2. Associations between Changes in SRH and Depressive Symptoms

In Table 3, crude (shown as Model 1) and multiple linear regression were used to calculate the association between changes in SRH and prenatal depressive symptoms. A decline in SRH ratings was significantly associated with higher depressive symptoms (B = 2.31, *p* = 0.035) (95% CI [0.15, 4.50]). Consistently low ratings were significantly associated with higher depressive symptoms (B = 4.10, *p* = 0.031) (95% CI [0.46, 8.16]). While only controlling for demographic characteristics in Model 2, a decline in SRH ratings was associated with higher prenatal depressive symptoms compared to those in the good+ category (B = 2.45, *p* = 0.025) (95% CI [0.46, 4.80]). Consistently low health ratings were not statistically associated with depressive symptoms in pregnancy compared to those in the good+ category (B = 4.01, *p* = 0.052) (95% CI [−0.08, 8.11]). Model 3 adjusted for demographic characteristics and the number of life stressors in the previous six months. In this model, consistently low ratings were statistically associated with higher depressive symptoms in pregnancy (B = 4.06, *p* = 0.030) (95% CI [0.08, 7.51]). Additionally, having more stressors was associated with higher prenatal depressive symptoms (B = 0.69, *p* = 0.002) (95% CI [0.32, 1.08]).

Model 1, displayed in Table 4, estimated the crude association between changes in SRH and postpartum depression at six weeks. Consistently low ratings were associated with higher depressive symptoms at six weeks postpartum (B = 3.87, *p* = 0.047) (95% CI [0.06, 7.83]). A decline in SRH ratings was not significantly associated with postpartum depressive symptoms (B = −0.26, *p* = 0.777) (95% CI [−2.19, 1.83]). While controlling for prenatal depressive symptoms in Model 2, a decline in SRH rating was statistically associated with fewer postpartum depressive symptoms (B = −1.79, *p* = 0.045) (95% CI [−3.54, 0.01]). Consistently low ratings were not associated with postpartum depressive symptoms (B = 1.49, *p* =0.222) (95% CI [−1.07, 4.06]). More depressive symptoms in pregnancy were statistically associated with more depressive symptoms at six weeks postpartum (B = 0.67, *p* = 0.001) (95% CI [0.51, 0.80]). In Model 3, while controlling for prenatal depressive symptoms and demographic characteristics, higher prenatal depressive symptoms were positively associated with more depressive symptoms (B = 0.66, *p* = 0.001) (95% CI [0.50, 0.80]). There was no significant association between postpartum depressive symptoms among women whose SRH declined (B = −1.74, *p* = 0.060) (95% CI [−3.75, 0.12]) or among women who reported consistently low ratings (B = 1.40, *p* = 0.267) (95% CI [−1.06, 4.02]). Model 4 controlled for demographic variables, prenatal depressive symptoms, and the number of life stressors. Higher depressive symptoms in pregnancy were statistically associated with higher depressive symptoms at six weeks post-childbirth (B *=* 0.62, *p* = 0.001) (95% CI [0.46, 0.77]). After controlling for prenatal depressive symptoms and demographic characteristics, consistently low and declined SRH ratings were not significantly associated with postpartum depressive symptoms. 

### 3.3. Associations between Changes in SRH and Anxiety Symptoms

Table 5 displays the results from crude (shown as Model 1) and adjusted linear regression models examining the association between SRH and prenatal anxiety symptoms. Consistently low SRH ratings were statistically associated with higher anxiety symptoms in pregnancy (B = 6.43, *p* = 0.036) (95% CI [−0.07, 2.72]). A decline in SRH ratings was statistically associated with higher prenatal anxiety symptoms (B *=* 7.29, *p* = 0.004) (95% CI [2.98, 12.14]). While controlling for demographic characteristics in Model 2, a decline in SRH rating was significantly associated with higher prenatal anxiety symptoms (B = 7.49, *p* = 0.004) (95% CI [3.24, 12.62]). Consistently low SRH ratings were not associated with anxiety symptoms in pregnancy (B = 6.03, *p* = 0.078) (95% CI [−1.14, 12.60]). Model 3 controlled for demographic characteristics and the number of stressors. A decline in SRH ratings was associated with higher prenatal anxiety symptoms (B = 6.99, *p* = 0.005) (95% CI [2.37, 11.88]). More stressors was associated with higher anxiety symptoms in pregnancy (B = 0.89, *p* = 0.010) (95% CI [0.19, 1.65]). Consistently low SRH ratings were not associated with prenatal anxiety symptoms (B = 6.10, *p* = 0.055) (95% CI [−0.006, 12.76]).

Regression models evaluating the association between SRH and postpartum anxiety symptoms at six weeks are shown in Table 6. Model 1 shows the crude associations between SRH and postpartum anxiety symptoms. Consistently low SRH ratings were statistically associated with higher postpartum anxiety symptoms (B = 7.03, *p* = 0.040) (95% CI [0.94, 14.31]). A decline in SRH ratings was not significantly associated with postpartum anxiety symptoms (B = 1.19, *p* = 0.583) (95% CI [−2.90, 6.13]). After controlling for prenatal anxiety symptoms in Model 2, more anxiety symptoms in pregnancy were associated with higher anxiety symptoms at six weeks postpartum (B = 0.63, *p* = 0.001) (95% CI [0.50, 0.77]). A decline in SRH ratings was associated with fewer postpartum anxiety symptoms (B = −3.35, *p* = 0.030) (95% CI [−6.42, −0.21]). Consistently low SRH ratings were not associated with postpartum anxiety (B = 3.15 *p* = 0.192) (95% CI [−1.36, 8.44]). Model 3 controlled for demographic characteristics and prenatal anxiety symptoms. Higher anxiety symptoms in pregnancy were associated with higher anxiety symptoms at six weeks post-childbirth (B = 0.63, *p* = 0.001) (95% CI [0.50, 0.77]). Consistently low (B = 2.65, *p* = 0.258) (95% CI [−1.40, 7.46]) and declined SRH ratings (B *=* −3.27, *p* = 0.055) (95% CI [−6.63, −0.01]) were not associated with postpartum anxiety symptoms. Model 4 controlled for prenatal anxiety symptoms, number of stressors, and demographic characteristics. Higher prenatal anxiety symptoms in pregnancy, on average, were significantly associated with higher postpartum anxiety symptoms (B = 0.61, *p* = 0.001) (95% CI [0.48, 0.76]). A decline in SRH ratings was statistically associated with postpartum anxiety symptoms (B = −3.44, *p* = 0.042) (95% CI [−6.62, −0.14]). After controlling for demographic characteristics, number of stressors, and prenatal anxiety symptoms, consistently low ratings were not associated with postpartum anxiety symptoms.

## 4. Discussion

Latina women are part of an increasing Latino population in the U.S. but are disproportionally disadvantaged during the perinatal period. Low SRH ratings and depressive and anxiety symptoms have been reported among this population. Currently, studies have focused on SRH ratings as the outcome variable or on depressive and anxiety symptoms as independent factors when investigating SRH ratings [21,22]. Few studies have investigated changes in SRH, and the association with perinatal depressive and anxiety symptoms remains unclear. This study aimed to assess the changes in SRH, from preconception to pregnancy, and its association with anxiety and depressive symptoms during the perinatal period in a sample of Latina women.

To our knowledge, this is the first study to examine whether changes in SRH ratings among Latina women were associated with perinatal anxiety and depressive symptoms in pregnancy and at six weeks post-childbirth. Although we expected that Latina women with a decline and consistently low SRH ratings would report more anxiety and depressive symptoms over time compared to the good+ group, we found partial support of our hypotheses. We found that life stressors were positively associated with depressive and anxiety symptoms in pregnancy. We also found that while controlling for demographic characteristics and life stressors, consistently low SRH was significantly associated with significantly higher prenatal depressive symptoms than good+ SRH, but not postpartum symptoms. A possible explanation is the life stressors weighed heavily on women, increasing their risk of depressive symptoms. Studies with perinatal women have found that stressors can impact a mother’s mental health and increase depressive symptoms [14,51]. In addition, this population experiences diverse stressors that can eventually affect their mental health [29,30]. Indeed, Table 2 shows that adding number of life stressors to the model eliminated the effect of a decline in SRH but increased the significance of a consistently low SRH on depressive symptoms in pregnancy. It is possible that pregnancy-related stressors not captured here may have negatively affected how women perceive their self-health, increasing their risk for depressive symptoms. This study assessed individual-level stressors and did not account for other stressors, such as community-level factors, that can occur during pregnancy. Others have found that depressive symptoms decline post-childbirth, and women’s pregnancy-related concerns level off after pregnancy [52,53]. This course of symptom improvement may explain the lack of effect of low SRH on postpartum depressive symptoms in our sample. 

While anxiety symptoms improved from pregnancy to six weeks postpartum, we also found that when controlling for demographic characteristics and life stressors, a decline in SRH from preconception to gestation was significantly and positively associated with prenatal and postpartum anxiety symptoms compared to good+ SRH. There was also a significant and positive association between the number of life stressors and prenatal anxiety symptoms, which supports our previous findings [54]. Interestingly, life stressors were not significantly associated with anxiety symptoms at six weeks postpartum. A possible explanation is that stressors may have had a more proximal effect on anxiety, thus having a waning effect postpartum when stressors that occurred within six months of pregnancy may have been perceived as distal or less impactful. Other studies have found that maternal anxiety increases the extent to which a mother perceives stress during pregnancy, but decreases as a mother is less anxious about her newborn child than while pregnant [55,56]. However, the effect of a decline in SRH persisted over time, suggesting that adverse effects of perceived health in pregnancy have a lasting consequence on women’s mental health. Interestingly, the findings on the effects of SRH on anxiety are equivocal. While, to our knowledge, no other study has examined the association between changes in SRH and perinatal anxiety symptoms, one study found that SRH was not associated with anxiety in pregnancy [57]. A possible explanation for our findings is that this is a vulnerable group of women who may have experienced stressors related to pregnancy, giving birth in the U.S., or social determinants of health. Therefore, future studies should assess those factors to ascertain better why a decline in SRH is associated with perinatal anxiety in Latinas.

The study’s findings contribute to the field of perinatal mental health and Latinas, such as comparing changes in SRH (i.e., decline, consistently low, or good+), which enabled us to see that the majority of our sample was part of the good+ group with very few belonging to the consistently low SRH rating and decline group. However, there are several limitations that must be addressed. First, we assessed changes in SRH from before pregnancy to pregnancy based on a one-item rating response without a given specific window of time. Therefore, there could be substantial variability in how women in the study interpreted the question and evaluated their preconception health, possibly resulting in biased reporting. Second, the sample size was small, limiting generalizability. Thus, future studies should use a larger sample of Latina women to improve statistical power. Third, the sample was drawn from one medical center, which inherently limits generalizability on all Latinas and increases potential bias. Fourth, we did not assess immigrant status or country of origin limiting our ability to determine differences due to these demographic characteristics. Therefore, further studies should collect data on the country of origin to determine its effects, if any, on SRH and perinatal mental health among Latinas. Fifth, all the data were self-reported, increasing the risk of reporter bias. Related, we did not confirm depression or anxiety status. Further studies should include a diagnostic measure of depression and anxiety. Additionally, we could not compute the internal consistency for the EPDS, STAI-T, and LES measures because the data provided for this secondary analysis did not include individual items. Future studies should assess the internal consistency when investigating this study population. Lastly, we did not assess health conditions that may have negatively affected SRH in pregnancy. While this was a relatively healthy sample, additional future studies should capture how new and pre-existing health conditions affect SRH and perinatal mental health (removed for blind review).

## 5. Conclusions

To our knowledge, this study is the first to investigate changes in SRH from preconception to pregnancy among a sample of perinatal Latina women to determine associations with depressive and anxiety symptoms. Our study showed that a decline in SRH ratings was statistically associated with more anxiety symptoms in pregnancy. Additionally, a consistently low SRH rating was associated with higher depressive symptoms in pregnancy. These findings highlight the urgency to identify risk factors for poor perinatal mental health among Latinos, who experience high psychosocial distress that negatively affects their mental health [58,59,60,61]. 

## Figures and Tables

**Table 1:** Self-Reported Demographic and Health Characteristics of Participating Latina Women.

Characteristic	N = 153	
Age (years), mean (±SD)	26.88	5.41
Education Level, n (%)		
H.S. or less	138	90.2
College or more	16	9.8
Family Income, n (%)		
Less than USD 10,000	22	14.4
USD 10,001–20,000	60	39.2
USD 20,001–30,000	43	28.1
USD 30,001	28	18.3
Number of life stressors, mean (±SD)	2.41	1.78
Self-rated health (before pregnancy), n (%)		
Excellent	31	20.3
Very good	25	16.3
Good	71	46.4
Fair	24	15.7
Poor	2	1.3
Self-rated health (since pregnancy), n (%)		
Excellent	18	11.8
Very good	20	13.1
Good	82	53.6
Fair	30	19.6
Poor	3	2.0
Change in self-rated health, n (%)		
Consistently low ^a^	12	7.8
Decline ^b^	21	13.7
Good+ ^c^	120	78.4

H.S.: high school; SD: standard deviation. ^a^ Consistently low describes women who consistently reported their health status as “fair” or “poor” before pregnancy and since pregnancy. ^b^ Decline describes women who reported their health status as “excellent”, “very good”, or “good” before pregnancy but “fair” or “poor” since pregnancy. ^c^: Good+ refers to all other women in the analytical sample.

**Table 2:** Results from the Wilcoxon Signed Rank Test Regarding the Pretest-Posttest of Depressive Scores, Anxiety Scores, and SRH.

	Prenatal			Postpartum			z-Score	*p*-Value
	Median	Mean	SD	Median	Mean	SD		
EPDS ^a^	7.00	6.71	4.64	4.50	5.58	4.74	−3.325	0.001
STAI ^b^	36.00	35.91	9.21	30.00	32.49	8.80	−5.215	<0.001
	Median							
Pre-pregnancy ^c^-Pregnancy ^d^	3.00						−3.125	0.001

Abbreviations: EPDS—Edinburg Postnatal Depression Scale. STAI-T—State-Trait Anxiety Inventory-Trait subscale. S.D.—Standard Deviation. ^a^ Based on 153 mothers. ^b^ Based on 142 mothers. ^c^ Based on 153 mothers. ^d^ Based on 153 mothers.

**Table 3:** Crude and Multiple Adjusted Linear Regression Models Examining Associations between Changes in Self-Rated Health and Prenatal Depressive Symptoms.

	Model 1			Model 2			Model 3		
	B	95% CI	*p*	B	95% CI	*p*	B	95% CI	*p*
SRH									
Consistently low	4.10	0.46–8.16	0.031	4.01	−0.080–8.11	0.052	4.06	0.08–7.51	0.030
Decline	2.31	0.15–4.50	0.035	2.45	0.46–4.80	0.025	2.07	−0.25–4.31	0.078
Good+	REF	REF	REF	REF	REF	REF	REF	REF	REF
Education level									
H.S. or less	-		-	−1.94	−4.03–0.40	0.085	−1.59	−3.73–0.75	0.176
College and more	-	-	-	REF	REF	REF	REF	REF	REF
Family income		-							
Less than USD 10,000	-	-	-	1.00	−1.54–3.84	0.466	0.76	−1.67–3.21	0.558
USD 10,001–20,000	-	-	-	−0.17	−2.18–1.59	0.845	−0.03	−1.66–1.64	0.969
USD 20,001–30,000	-	-	-	−0.40	−2.39–1.32	0.642	−0.02	−1.74–1.70	0.988
USD 30,001	-	-	-	REF	REF	REF	REF	REF	REF
Age, years	-	-	-	0.02	−0.12–0.16	0.790	0.03	−0.09–0.16	0.587
Number of stressors	-	-	-	-	-	-	0.69	0.32–1.08	0.002

Notes: B = Coefficient; 95% CI: Confidence Intervals. Model 1: crude model examining the association between self-reported health and prenatal depressive symptoms. Model 2: adjusted for age, education, and family income. Model 3: adjusted for age, education, family income, and number of life stressors. Abbreviations: SRH—Self-rated health, H.S.—high school.

**Table 4:** Crude and Multiple Adjusted Linear Regression Models Examining Associations between Change in Self-Rated Health and Postpartum Depressive Symptoms at Six Weeks.

	Model 1			Model 2			Model 3			Model 4		
	B	95% CI	*p*	B	95% CI	*p*	B	95% CI	*p*	B	95% CI	*p*
SRH												
Consistently low	3.87	0.06–7.83	0.047	1.49	−1.07–0.06	0.222	1.40	−1.06–4.02	0.267	1.52	−0.76–4.13	0.230
Decline	−0.26	−2.19–1.83	0.777	−1.79	−3.54–0.01	0.045	−1.74	−3.75–0.12	0.060	−1.84	−3.67–0.12	0.061
Good+	REF	REF	REF	REF	REF	REF	REF	REF	REF	REF	REF	REF
Prenatal depressive symptoms				0.67	0.51–0.80	0.001	0.66	0.50–0.80	0.001	0.62	0.46–0.12	0.001
Education level												
H.S. or less	-	-	-	-	-	-	−0.11	−2.66–2.15	0.929	−0.01	−2.36–2.30	0.998
College and more	-	-	-	-	-	-	REF	REF	REF	REF	REF	REF
Family income												
Less than USD 10,000	-	-	-	-	-	-	−1.22	−3.33–0.85	0.286	−1.29	−3.59–0.92	0.245
USD 10,001–20,000	-	-	-	-	-	-	−0.63	−2.65–1.18	0.523	−0.54	−2.42–1.30	0.559
USD 20,001–30,000	-	-	-	-	-	-	−1.58	−3.39–0.22	0.093	−1.43	−3.32–0.49	0.129
USD 30,001	-	-	-	-	-	-	REF	REF	REF	REF	REF	REF
Age, years	-	-	-	-	-	-	0.03	−0.09–0.14	0.649	0.03	−0.08–0.15	0.542
Number of stressors	-	-	-	-	-	-	-	-	-	0.30	−0.03–0.68	0.094

Notes: B = Coefficient; 95% CI: Confidence Intervals. Model 1: crude model examining the association between self-reported health and postpartum depressive symptoms at six weeks. Model 2: adjusted for prenatal depressive symptoms. Model 3: adjusted for age, education, family income, and prenatal depressive symptoms. Model 4: adjusted for age, education, family income, prenatal depressive symptoms, and number of life stressors. Abbreviations: SRH—Self-rated health, H.S.—high school.

**Table 5:** Crude and Multiple Adjusted Linear Regression Models Examining Associations between Change in Self-Rated Health and Prenatal Anxiety Symptoms.

	Model 1			Model 2			Model 3		
	B	95% CI	*p*	B	95% CI	*p*	B	95% CI	*p*
SRH									
Consistently low	6.43	−0.07–2.72	0.036	6.03	−1.14–12.60	0.078	6.10	−0.006–12.76	0.055
Decline	7.29	2.98–12.14	0.004	7.49	3.237–12.62	0.004	6.99	2.37–11.88	0.005
Good+	REF	REF	REF	REF	REF	REF	REF	REF	REF
Education level									
H.S. or less	-	-	-	−2.43	−6.77–2.11	0.309	−1.97	−6.58–2.53	0.373
College and more	-	-	-	REF	REF	REF	REF	REF	REF
Family income		-							
Less than USD 10,000	-	-	-	1.98	−2.95–7.11	0.401	1.67	−3.11–6.17	0.478
USD 10,001–20,000	-	-	-	−0.84	−4.80–3.33	0.685	−0.66	−4.52–3.06	0.743
USD 20,001–30,000	-	-	-	−2.04	−5.39–1.58	0.263	−1.53	−4.98–1.95	0.391
USD 30,001	-	-	-	REF	REF	REF	REF	REF	REF
Age, years	-	-	-	−0.03	−0.33–0.25	0.841	−0.01	−0.28–0.27	0.939
Number of stressors	-	-	-	-	-	-	0.89	0.19–1.65	0.010

Notes: B = Coefficient; 95 % CI: Confidence Intervals. Model 1: crude model examining the association between self-rated health. Model 2 adjusted for age, education, and family income. Model 3: adjusted for age, education, family income, and number of life stressors. Abbreviations: SRH—Self-rated health, H.S.—high school.

**Table 6:** Crude and Multiple Adjusted Linear Regression Models Examining Associations between Change in Self-Rated Health and Postpartum Anxiety Symptoms at Six Weeks.

	Model 1			Model 2			Model 3			Model 4		
	B	95% CI	*p*	B	95% CI	*p*	B	95% CI	*p*	B	95% CI	*p*
SRH												
Consistently low	7.03	0.94–14.31	0.040	3.15	−1.36–8.44	0.192	2.65	−1.40–7.46	0.258	2.76	−1.29–7.25	0.213
Decline	1.19	−2.90–6.13	0.583	−3.35	−6.42–0.21	0.030	−3.27	−6.63–0.01	0.055	−3.44	−6.62–0.14	0.042
Good+	REF	REF	REF	REF	REF	REF	REF	REF	REF	REF	REF	REF
Prenatal anxiety symptoms				0.63	0.50–0.77	0.001	0.63	0.50–0.77	0.001	0.61	0.48–0.76	0.001
Education level												
H.S. or less	-	-	-	-	-	-	−0.42	−5.05–3.65	0.853	−0.20	−5.06–4.15	0.922
College and more	-	-	-	-	-	-	REF	REF	REF	REF	REF	REF
Family income		-										
Less than USD 10,000	-	-	-	-	-	-	−1.88	−5.90–1.92	0.361	−2.03	−6.33–1.69	0.342
USD 10,001–20,000	-	-	-	-	-	-	0.06	−3.47–3.24	0.973	0.20	−3.57–3.34	0.930
USD 20,001–30,000	-	-	-	-	-	-	−2.17	−5.74–1.45	0.218	−1.94	−5.66–1.29	0.289
USD 30,001	-	-	-	-	-	-	REF	REF	REF	REF	REF	REF
Age, years	-	-	-	-	-	-	0.14	−0.07–0.32	0.171	0.15	−0.06–0.35	0.148
Number of stressors	-	-	-	-	-	-	-	-	-	0.47	−0.14–1.16	0.158

Notes: B = Coefficient; CI: Confidence Intervals. Model 1: crude model examining the association between self-rated health and postpartum anxiety symptoms at six weeks. Model 2: adjusted for prenatal anxiety symptoms. Model 3: adjusted for age, education, family income, and prenatal anxiety symptoms. Model 4: adjusted for age, education, family income, number of life stressors, and prenatal anxiety symptoms. Abbreviations: SRH—Self-rated health, H.S.—high school.

## Data Availability

The data presented in this study are available upon request from the corresponding author.

## References

[1] Alhusen J.L., Alvarez C. (2016). Perinatal Depression: A Clinical Update. Nurse Pract..

[2] Fawcett E.J., Fairbrother N., Cox M.L., White I.R., Fawcett J.M. (2019). The Prevalence of Anxiety Disorders During Pregnancy and the Postpartum Period: A Multivariate Bayesian Meta-Analysis. J. Clin. Psychiatry.

[3] American College of Obstetricians and Gynecologists Committee (2015). Committee Opinion No. 630. Screening for Perinatal Depression. Obstet. Gynecol..

[4] Gavin N.I., Gaynes B.N., Lohr K.N., Meltzer-Brody S., Gartlehner G., Swinson T. (2005). Perinatal Depression: A Systematic Review of Prevalence and Incidence. Obstet. Gynecol..

[5] Woody C.A., Ferrari A.J., Siskind D.J., Whiteford H.A., Harris M.G. (2017). A Systematic Review and Meta-Regression of the Prevalence and Incidence of Perinatal Depression. J. Affect. Disord..

[6] Kieffer E.C., Caldwell C.H., Welmerink D.B., Welch K.B., Sinco B.R., Guzmán J.R. (2013). Effect of the Healthy Moms Lifestyle Intervention on Reducing Depressive Symptoms among Pregnant Latinas. Am. J. Community Psychol..

[7] Lucero N.B., Beckstrand R.L., Callister L.C., Sanchez Birkhead A.C. (2012). Prevalence of Postpartum Depression among Hispanic Immigrant Women. J. Am. Acad. Nurse Pract..

[8] Ceballos M., Wallace G., Goodwin G. (2016). Postpartum Depression among African-American and Latina Mothers Living in Small Cities, Towns, and Rural Communities. J. Racial Ethn. Health Disparities.

[9] Preciado A., D’Anna-Hernandez K. (2017). Acculturative Stress Is Associated with Trajectory of Anxiety Symptoms During Pregnancy in Mexican-American Women. J. Anxiety Disord..

[10] Garcia E.R., Yim I.S. (2017). A Systematic Review of Concepts Related to Women’s Empowerment in the Perinatal Period and Their Associations with Perinatal Depressive Symptoms and Premature Birth. BMC Pregnancy Childbirth.

[11] Dennis C.L., Falah-Hassani K., Shiri R. (2017). Prevalence of Antenatal and Postnatal Anxiety: Systematic Review and Meta-Analysis. Br. J. Psychiatry.

[12] Enatescu V.R., Enatescu I., Craina M., Gluhovschi A., Papava I., Romosan R., Marian C., Oprea A., Bernad E. (2014). State and Trait Anxiety as a Psychopathological Phenomenon Correlated with Postpartum Depression in a Romanian Sample: A Pilot Study. J. Psychosom. Obstet. Gynecol..

[13] Martini J., Wittich J., Petzoldt J., Winkel S., Einsle F., Siegert J., Hoefler M., Beesdo-Baum K., Wittchen H.U. (2013). Maternal Anxiety Disorders Prior to Conception, Psychopathology During Pregnancy and Early Infants’ Development: A Prospective-Longitudinal Study. Arch. Women’s Ment. Health.

[14] Field T. (2018). Postnatal Anxiety Prevalence, Predictors and Effects on Development: A Narrative Review. Infant Behav. Dev..

[15] Lamers F., van Oppen P., Comijs H.C., Smit J.H., Spinhoven P., van Balkom A.J., Nolen W.A., Zitman F.G., Beekman A.T., Penninx B.W. (2011). Comorbidity Patterns of Anxiety and Depressive Disorders in a Large Cohort Study: The Netherlands Study of Depression and Anxiety (Nesda). J. Clin. Psychiatry.

[16] Barcelona de Mendoza V., Harville E., Theall K., Buekens P., Chasan-Taber L. (2016). Effects of Acculturation on Prenatal Anxiety among Latina Women. Arch. Women’s Ment. Health.

[17] Idler E.L., Benyamini Y. (1997). Self-Rated Health and Mortality: A Review of Twenty-Seven Community Studies. J. Health Soc. Behav..

[18] Bombak A.E. (2013). Self-Rated Health and Public Health: A Critical Perspective. Front. Public Health.

[19] Brown T.H., Richardson L.J., Hargrove T.W., Thomas C.S. (2016). Using Multiple-Hierarchy Stratification and Life Course Approaches to Understand Health Inequalities: The Intersecting Consequences of Race, Gender, Ses, and Age. J. Health Soc. Behav..

[20] Chandola T., Jenkinson C. (2000). Validating Self-Rated Health in Different Ethnic Groups. Ethn. Health.

[21] Schytt E., Hildingsson I. (2011). Physical and Emotional Self-Rated Health among Swedish Women and Men During Pregnancy and the First Year of Parenthood. Sex. Reprod. Healthc..

[22] Semasaka J.P., Krantz G., Nzayirambaho M., Munyanshongore C., Edvardsson K., Mogren I. (2016). Self-Reported Pregnancy-Related Health Problems and Self-Rated Health Status in Rwandan Women Postpartum: A Population-Based Cross-Sectional Study. BMC Pregnancy Childbirth.

[23] Mechakra-Tahiri S., Zunzunegui M.V., Seguin L. (2007). Self-Rated Health and Postnatal Depressive Symptoms among Immigrant Mothers in Quebec. Women Health.

[24] Orr S.T., Blazer D.G., James S.A., Reiter J.P. (2007). Depressive Symptoms and Indicators of Maternal Health Status During Pregnancy. J. Women’s Health.

[25] Schytt E., Waldenstrom U. (2007). Risk Factors for Poor Self-Rated Health in Women at 2 Months and 1 Year after Childbirth. J. Women’s Health.

[26] Sonkusare S., Adinegara Hebbar S. (2007). Mental Health, Pregnancy and Self-Rated Health in Antenatal Women Attending Primary Health Clinics. Med. J. Malays..

[27] Lara-Cinisomo S., Swinford C., Massey D., Hardt H. (2018). Diabetes, Prenatal Depression, and Self-Rated Health in Latina Mothers. Diabetes Spectr..

[28] Gheorghe M., Varin M., Wong S.L., Baker M., Grywacheski V., Orpana H. (2020). Symptoms of Postpartum Anxiety and Depression among Women in Canada: Findings from a National Cross-Sectional Survey. Can. J. Public Health.

[29] Lara-Cinisomo S., Girdler S.S., Grewen K., Meltzer-Brody S. (2016). A Biopsychosocial Conceptual Framework of Postpartum Depression Risk in Immigrant and U.S.-Born Latina Mothers in the United States. Women’s Health Issues.

[30] Rosenthal L., Earnshaw V.A., Lewis T.T., Reid A.E., Lewis J.B., Stasko E.C., Tobin J.N., Ickovics J.R. (2015). Changes in Experiences with Discrimination across Pregnancy and Postpartum: Age Differences and Consequences for Mental Health. Am. J. Public Health.

[31] D’Anna L.H., Ponce N.A., Siegel J.M. (2010). Racial and Ethnic Health Disparities: Evidence of Discrimination’s Effects across the Sep Spectrum. Ethn. Health.

[32] Benjamins M.R., Hirschman J., Hirschtick J., Whitman S. (2012). Exploring Differences in Self-Rated Health among Blacks, Whites, Mexicans, and Puerto Ricans. Ethn. Health.

[33] Idler E.L., Angel R.J. (1990). Self-Rated Health and Mortality in the Nhanes-I Epidemiologic Follow-up Study. Am. J. Public Health.

[34] Pedersen C., Leserman J., Garcia N., Stansbury M., Meltzer-Brody S., Johnson J. (2016). Late Pregnancy Thyroid-Binding Globulin Predicts Perinatal Depression. Psychoneuroendocrinology.

[35] Ware J., Kosinski M., Keller S.D. (1996). A 12-Item Short-Form Health Survey: Construction of Scales and Preliminary Tests of Reliability and Validity. Med. Care.

[36] Kraus M.W., Adler N., Chen T.W. (2013). Is the Association of Subjective Ses and Self-Rated Health Confounded by Negative Mood? An Experimental Approach. Health Psychol..

[37] Sarason I.G., Johnson J.H., Siegel J.M. (1978). Assessing the Impact of Life Changes: Development of the Life Experiences Survey. J. Consult. Clin. Psychol..

[38] Harville E.W., Savitz D.A., Dole N., Herring A.H., Thorp J.M. (2009). Stress Questionnaires and Stress Biomarkers During Pregnancy. J. Women’s Health.

[39] Campos R.C., Holden R.R., Baleizão C., Caçador B., Fragata A.S. (2018). Self-Criticism, Neediness, and Distress in the Prediction of Suicide Ideation: Results from Cross-Sectional and Longitudinal Studies. J. Psychol..

[40] Grau J.M., Castellanos P., Smith E.N., Duran P.A., Silberman S., Wood L. (2017). Psychological Adjustment among Young Puerto Rican Mothers: Perceived Partner Support and the Moderating Role of Latino Cultural Orientation. J. Lat. Psychol..

[41] Zayas L.H., Jankowski K.R., McKee M.D. (2003). Prenatal and Postpartum Depression among Low-Income Dominican and Puerto Rican Women. Hisp. J. Behav. Sci..

[42] Cox J.L., Holden J.M., Sagovsky R. (1987). Detection of Postnatal Depression. Development of the 10-Item Edinburgh Postnatal Depression Scale. Br. J. Psychiatry.

[43] Carter E.A., Bond M.J., Wickham R.E., Barrera A.Z. (2019). Perinatal Depression among a Global Sample of Spanish-Speaking Women: A Sequential-Process Latent Growth-Curve Analysis. J. Affect. Disord..

[44] Lara-Cinisomo S., D’Anna-Hernandez K., Fujimoto E.M., Pedersen C.A. (2019). Exploring Associations between Perinatal Depression, Anxiety, and Urinary Oxytocin Levels in Latinas. Arch. Women’s Ment. Health.

[45] Dolbier C.L., Rush T.E., Sahadeo L.S., Shaffer M.L., Thorp J., Investigators Community Child Health Network (2013). Relationships of Race and Socioeconomic Status to Postpartum Depressive Symptoms in Rural African American and Non-Hispanic White Women. Matern. Child Health J..

[46] Alvarado R., Jadresic E., Guajardo V., Rojas G. (2015). First Validation of a Spanish-Translated Version of the Edinburgh Postnatal Depression Scale (Epds) for Use in Pregnant Women. A Chilean Study. Arch. Women’s Ment. Health.

[47] Spielberger C.D., Gorsuch R., Lushene R., Vagg P.R., Jacobs G.A. (1983). Manual for the State-Trait Anxiety Inventory.

[48] Kimport E.R., Hartzell E. (2015). Clay and Anxiety Reduction: A One-Group, Pretest/Posttest Design with Patients on a Psychiatric Unit. Art Ther..

[49] Dennis C.L., Coghlan M., Vigod S. (2013). Can We Identify Mothers at-Risk for Postpartum Anxiety in the Immediate Postpartum Period Using the State-Trait Anxiety Inventory?. J. Affect. Disord..

[50] Julian L.J. (2011). Measures of Anxiety: State-Trait Anxiety Inventory (Stai), Beck Anxiety Inventory (Bai), and Hospital Anxiety and Depression Scale-Anxiety (Hads-a). Arthritis Care Res..

[51] Tarimo B.B., Law H.C., Tao D., Pastrana-Mena R., Kanzok S.M., Buza J.J., Dinglasan R.R. (2018). Paraquat-Mediated Oxidative Stress in Anopheles Gambiae Mosquitoes Is Regulated by an Endoplasmic Reticulum (Er) Stress Response. Proteomes.

[52] Akinbode T.D., Pedersen C., Lara-Cinisomo S. (2021). The Price of Pre-Adolescent Abuse: Effects of Sexual Abuse on Perinatal Depression and Anxiety. Matern. Child Health J..

[53] Heron J., O’Connor T.G., Evans J., Golding J., Glover V., ALSPAC Study Team (2004). The Course of Anxiety and Depression through Pregnancy and the Postpartum in a Community Sample. J. Affect. Disord..

[54] Razurel C., Kaiser B., Antonietti J.P., Epiney M., Sellenet C. (2017). Relationship between Perceived Perinatal Stress and Depressive Symptoms, Anxiety, and Parental Self-Efficacy in Primiparous Mothers and the Role of Social Support. Women Health.

[55] Huizink A.C., Menting B., De Moor M.H., Verhage M.L., Kunseler F.C., Schuengel C., Oosterman M. (2017). From Prenatal Anxiety to Parenting Stress: A Longitudinal Study. Arch. Women’s Ment. Health.

[56] Lutkiewicz K., Bieleninik L., Cieslak M., Bidzan M. (2020). Maternal-Infant Bonding and Its Relationships with Maternal Depressive Symptoms, Stress and Anxiety in the Early Postpartum Period in a Polish Sample. Int. J. Environ. Res. Public Health.

[57] Christian L.M., Iams J., Porter K., Leblebicioglu B. (2013). Self-Rated Health among Pregnant Women: Associations with Objective Health Indicators, Psychological Functioning, and Serum Inflammatory Markers. Ann. Behav. Med..

[58] Alhasanat D., Giurgescu C. (2017). Acculturation and Postpartum Depressive Symptoms among Hispanic Women in the United States: Systematic Review. Am. J. Matern. Child Nurs..

[59] Edwards L.M., Le H.N., Garnier-Villarreal M. (2021). A Systematic Review and Meta-Analysis of Risk Factors for Postpartum Depression among Latinas. Matern. Child Health J..

[60] Lara-Cinisomo S., Clark C.T., Wood J. (2018). Increasing Diagnosis and Treatment of Perinatal Depression in Latinas and African American Women: Addressing Stigma Is Not Enough. Women’s Health Issues.

[61] Schminkey D.L., Liu X., Annan S., Sawin E.M. (2019). Contributors to Health Inequities in Rural Latinas of Childbearing Age: An Integrative Review Using an Ecological Framework. Sage Open.

